# Transparency in Medicare Reimbursement: Help or Hindrance?

**DOI:** 10.6004/jadpro.2014.5.4.1

**Published:** 2014-07-01

**Authors:** Wendy H. Vogel

**Affiliations:** Ms. Vogel is a nurse practitioner at Wellmont Cancer Institute in Kingsport, Tennesee.

## Abstract

Welcome to the latest edition of JADPRO! It is my pleasure and honor to serve as an associate editor for this elite and unique journal. As one of the co-chairs of the meeting, I would like to invite you to attend the second JADPRO Live symposium to be held in sunny Orlando, Florida, this October 30 through November 2. The faculty lineup is superb, the content offerings are first rate, and you can earn up to 15 CE credits/contact hours. I am hoping to network with our entire readership there! You can find more information and learn how to register at www.apsho.org/jadprolive

**Transparency** 

When utilized in a business, humanities, or social contact, transparency is a concept that implies openness, communication, and accountability. It is a state that provides all relevant information freely and fully to the public.

In the spirit of transparency, the Centers for Medicare & Medicaid Services (CMS) recently released data on specific Medicare reimbursement claims. The Medicare Provider Utilization and Payment Data: Physician and Other Supplier public use database gives information on services and procedures provided to Medicare beneficiaries in the 2012 calendar year by physicians and other health-care professionals. Information on utilization, payment (allowed amount and Medicare payment), and submitted charges is organized by National Provider Identifier, Healthcare Common Procedure Coding System (HCPCS) code, and place of service.

The database has a searchable tool (https://data.cms.gov/utilization-and-payment-explorer) through which any health-care provider, including advanced practitioners (APs), can be scrutinized. Specialists can also be compared with respect to Medicare billings and reimbursement. For example, a search reveals that oncologists, ophthalmologists, and pathologists are among the highest paid specialists.

The information amassed in the database had been confidential since 1979. Those clamoring for its release claimed it would help identify waste and fraud. Advocates for the data release believe it will help health-care recipients identify the providers who deliver the highest quality and most efficient care.

## Database Limitations

There are many, however, who believe that these data are at best incomplete and at worst inaccurate, biased, without context, and unrepresentative of true Medicare reimbursement. There is a fear that the public will be increasingly confused and more distrustful of an already crippled health-care system.

The data show that $77 billion was paid to 880,000 US physicians in 2012 for just Medicare Part B (in oncology this means outpatient services such as lab tests, surgeries, chemotherapy and support medications, and office visits). However, this does not reflect the billing health-care professional’s cost of care, including such factors as chemotherapy drugs, equipment, staff, and overhead. It is feared that the highest billers (more than $3 million in that year) may be scrutinized more closely.

## The Oncology Setting

What does this mean for the oncology patient? Dealing with the stress of cancer and cancer treatment is enough without the additional anxiety of this controversial move. The public perception may have negative consequences when Congress makes decisions about health-care payments, sequester cuts, etc., potentially affecting recruitment of professionals to the oncology field, access to care, and the cost of care.

I examined my own data and that of my AP and physician colleagues. I was able to see how many visits I had billed Medicare for by the HCPCS code. I learned that my most commonly billed code was 99214. I see what was billed to Medicare, what Medicare "allowed," and what the average payment was for this code. My data were comparable to those of the two physicians in our practice.

Oncologists who own their own chemotherapy infusion suites will have much more information available, such as how many injections of growth factors were prescribed. If the provider bills for diagnostic testing such as chest x-rays, that information will be available as well. Because cancer is more common in the Medicare population, oncology practitioners will naturally have a higher percentage of Medicare reimbursements as a result. Therefore, the comparison between specialties, such as gynecologists and oncologists, is not necessarily relevant.

## Implications for the Advanced Practitioner

If you are billing "incident-to," the physician that you bill under may appear to have more claims than his/her peers, and the AP’s work is not recognized. If the AP bills Medicare independently, the AP can be benchmarked against his/her colleagues, including physicians. The AP or physician could potentially utilize the data when making decisions about joining a practice. For example, one could compare the billings of the providers in a practice and benchmark this against one’s own data or those of another practice.

I encourage you to go to the searchable tool, search for yourself, and verify the accuracy of your data. Unfortunately, no provider was able to view or verify the information before it was made public. Many have reported their data to be inaccurate and not reflective of their true practice. For example, one provider’s name may be associated with billing for an entire practice, yet this would not be apparent to anyone examining the data.

## Unanswered Questions

There are many unanswered questions about this data release. What do you do if your information is inaccurate? How will this impact the patient-provider relationship? How will these data be used to identify fraud and waste? Who will be responsible for pursuing these crimes, and what will be the cost to taxpayers? What are the moral and ethical implications? Is it an invasion of my privacy for you to know what I billed Medicare for in 2012? How does this information benefit the oncology patient? How will it affect AP practice? Will the public still see us as cost-effective providers? How can we help our patients interpret this information?

Perhaps we can discuss this and so much more when we meet in Orlando at JADPRO Live! 

